# 1p36 Deletion Syndrome and the Aorta: A Report of Three New Patients and a Literature Review

**DOI:** 10.3390/jcdd8110159

**Published:** 2021-11-19

**Authors:** Valentina Lodato, Valeria Orlando, Viola Alesi, Silvia Di Tommaso, Mario Bengala, Giovanni Parlapiano, Elisa Agnolucci, Marianna Cicenia, Federica Calì, Maria Cristina Digilio, Fabrizio Drago, Antonio Novelli, Anwar Baban

**Affiliations:** 1 The European Reference Network for Rare, Low Prevalence and Complex Diseases of the Heart-ERN GUARD-Heart, Pediatric Cardiology and Arrhythmia/Syncope Units, Bambino Gesù Children Hospital and Research Institute, IRCCS, 00165 Rome, Italy; valentina.lodato@opbg.net (V.L.); elisa.agnolucci@opbg.net (E.A.); marianna.cicenia@opbg.net (M.C.); federica.cali@opbg.net (F.C.); fabrizio.drago@opbg.net (F.D.); 2 Laboratory of Medical Genetics, Translational Cytogenomics Research Unit, Bambino Gesù Children Hospital and Research Institute, IRCCS, 00165 Rome, Italy; valeria.orlando@opbg.net (V.O.); viola.alesi@opbg.net (V.A.); silviaditommaso@opbg.net (S.D.T.); giovanni.parlapiano@opbg.net (G.P.); antonio.novelli@opbg.net (A.N.); 3 Laboratory of Medical Genetics, Tor Vergata Hospital, 00133 Rome, Italy; mario.bengala@ptvonline.it; 4 Genetics and Rare Diseases Division, Bambino Gesù Children Hospital and Research Institute, IRCCS, 00165 Rome, Italy; mcristina.digilio@opbg.net

**Keywords:** 1p36 deletion syndrome, heart, aortic dilatation, *SKI*

## Abstract

Background: Monosomy 1p36 syndrome is now considered the most common terminal deletion syndrome, with an estimated incidence of 1 in 5000. Cardiac involvement is well described in the literature mainly in terms of congenital heart defects (CHDs) and cardiomyopathies (CMPs). Few data in the literature describe the potential progressive nature of aortic dilatation (root and ascending aorta) in 1p36 deletion syndrome. *SKI* harboured in the deleted region might play a predisposing factor for this aspect. Methods: we reviewed the aortic aspect both in the literature and in our cohort, where major attention to the aortic abnormalities was given through dedicated echocardiographic measurements even in previously screened individuals. Results: aortic involvement in 1p36 deletion syndrome was described in the literature three times within the CHD context. We observed three additional patients from our cohort (three out of nine patients) with aortic dilatation. All patients with dilated aorta had *SKI* haploinsufficiency within the deleted region. Conclusions: at long-term outcome and with a growing population of this rare disease, this association (1p36 deletion and aortic dilatation) might represent a major concern especially in terms of risk stratification and the potential need for specific management (conservative pharmacologic and eventually surgical) whenever indicated. The present study suggests the need for detailed multicentric studies and indication to periodic echocardiographic screening in addition to baseline tests, especially in individuals with deletions harbouring *SKI*.

## 1. Introduction

Chromosome 1p36 deletion syndrome, first described by Yunis et al. [1], is a common sub-telomeric microdeletion observed in humans. It results in a contiguous gene syndrome (OMIM #607872) characterised mainly by congenital anomalies and intellectual disability (ID) [2]. The incidence is approximately 1 in 5000 live births [3]. However, in the last decade, it seems more frequent probably due to increased diagnostic use of microarray analysis [4]. In 52%–67% of patients, it is caused by the heterozygous deletion of the distal chromosomal band on the short arm of chromosome 1. Less commonly, it can be caused by other rearrangements (interstitial deletions or complex rearrangements) [5].

Typical craniofacial dysmorphisms, developmental delay, and ID are the clinical signs observed in the majority of patients. However, it can be associated with multiple congenital anomalies: malformations of the central nervous system (88%), heart defects (71–75%), seizures (44–79%), skeletal anomalies (41%), vision problems, hearing loss, and other features. Increased knowledge has led to previous attempts at creating a genotype–phenotype correlation compared with the different sizes and positions of the 1p36 deletion. The phenotypic variability might be related to the involvement of specific genes located in the deleted chromosomal region [6].

The deletion ranged from 1.5 Mb to 10.5 Mb, and two critical regions are known: the distal critical region and proximal critical region [7]. The canonical distal critical region for 1p36 deletion syndrome is localised approximately 4 Mb from the 1p telomere. However, non-overlapping interstitial deletions involving the proximal region of 1p36 have also been shown to cause a similar phenotype. Some individuals have deletions of both the distal and proximal regions of 1p36. In such cases, the condition is often with worse prognosis [8,9]. Recently, Radio and colleagues described a third centromeric region in which *SPEN* haploinsufficiency represents a major contributor to the conditions associated with 1p36 deletions encompassing 1p36.21p36.13 [10].

1p36 deletions are associated with cardiovascular abnormalities, which are classically subdivided into congenital heart defects (CHDs) and cardiomyopathies (CMPs).

The most common CHDs described in the literature in 1p36 deletion syndrome are atrial and ventricular septal defects (ASD, VSD), patent ductus arteriosus (PDA), valvular anomalies, tetralogy of Fallot (TOF), and coarctation of the aorta (CoAo). Genes that may contribute to CHDs in 1p36 deletions are *DVL1*, *SKI*, *RERE*, *PDPN*, *SPEN*, *CLCNKA*, *ECE1*, *HSPG2*, *LUZP1,* and *WASPF2* [11].

Patients with CMP, are mostly represented in noncompact left ventricle (NCLV) and less frequently with end stage dilated cardiomyopathy (DCM). Previous studies suggested that genes that may lead to CMP in 1p36 deletions include *SKI*, *PRKCZ*, *PRDM16*, *RERE*, *UBE4B*, and *MASP2* [11].

Among the cardiovascular abnormalities, aortic involvement is potentially underestimated. Bicuspid aortic valve (BAV) and CoAo are classically and uncommonly reported to be within the CHD spectrum in terms of left-sided heart lesions. However, few reports described a potential predisposition to a progressive aortic dilatation in 1p36 deletion. Few data bring light to the genes of interest in this region that might play roles in leading to progressive aortic dilatation. One of these genes, *SKI*, is classically known to be related to Shprintzen–Goldberg syndrome (SGS). In fact, one of the major signs in this condition is the progressive aortic dilatation and risk of aneurysm (34%) [12], in addition to ASD, mitral valve prolapse, and vascular tortuosity [11]. Conservative medical management with angiotensin receptor blockers (ARBs) and beta blockers (β-blockers) in SGS should be considered in order to reduce hemodynamic stress since *SKI* is part of TGF-β pathway cascade. A reported risk of aortic dissection was previously described in the literature [12].

Taking in consideration all of these factors, we reviewed this specific aspect both in the literature and in our cohort, giving major attention to aortic dilatation in particular of the aortic root and ascending aorta, identifying this factor in (3/9) 30% of our cohort. 

## 2. Materials and Methods

We reviewed the medical records of patients with 1p36 deletion seen in our tertiary care centre. This is a single-centre, observational, both retro and prospective analysis. All data, including the cardiac diagnosis and surgical reports, were extracted from our cardiac database in the period from 2009 to 2020. The following parameters were collected: personal and family history, physical examination, anthropometric measurements, cardiac evaluation (including case notes, electrocardiogram, echocardiogram, catheterisation, and operative notes), screening for extracardiac malformations including cerebral ultrasound and/or magnetic resonance imaging (MRI), eye and hearing evaluations, and renal ultrasound. 

### 2.1. Literature Review

We performed a review of previous studies describing the co-existence of 1p36 deletion syndrome and aortic involvement in the paediatric population. We searched *PubMed* for published studies with no restriction on the date of publication and no restriction on the language, using the search terms “1p36 deletion” AND “aorta” AND “vascular changes” or “1p36 monosomy” AND “aortopathies” AND “aortic valve”. We included all types of studies, provided that the study population encompassed at least one patient with 1p36 deletion diagnosed and concomitant aortic disease. Full papers were carefully read and reconsidered according to the abovementioned criteria. Two investigators performed the search independently. The references of the selected papers were crosschecked with the same inclusion condition. Duplicates were removed.

### 2.2. Editorial Policies and Ethical Considerations

The study protocol conforms to ethical guidelines of the 1975 Declaration of Helsinki, as reflected in a priori approval by the Institution’s Human Research Committee. We confirm that informed consent was obtained from the parents of our probands. 

### 2.3. CMA (Chromosome Microarray Analysis) Techniques

All patients were analysed using an Array-CGH 4x180K (Agilent Technologies, Santa Clara, CA, USA) or Infinium CytoSNP-850K BeadChip (Illumina, San Diego, CA, USA) platform. Confirmation and segregation tests on patient’s and parents’ DNA were performed by fluorescence in situ hybridisation (FISH) analysis.

## 3. Results

**Figure 1:**
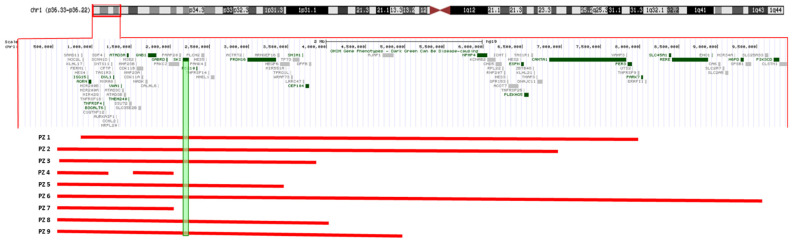
The extension of 1p36 deletions in our cohort showing sparing of *SKI* in patients 4 and 7. Both of these individuals have normal cardiac and aortic anatomy and function.

We report on nine patients harbouring a distal 1p36 deletion ranging from 1.2 to 9.9 Mb. Only one patient (ID 6) carried a more extensive deletion, 9.5 Mb in size, partially overlapping the proximal 1p36 deletion critical region (Figure 1). A segregation analysis revealed that the deletion arose “de novo” in all cases. Gender included five females and four males. The age at diagnosis ranged from 0 to 5 years old.

### Patients

Detailed clinical descriptions of our patient series are included in Supplementary Material.

## 4. Discussion

Monosomy 1p36 syndrome is now considered a common terminal deletion syndrome, with an estimated incidence of 1 in 5000. The disorder is due to the partial loss of material from the short arm of chromosome 1, being de novo in the majority of cases [7,13]. The 1p36 deletion syndrome phenotype is variable and major signs include craniofacial dysmorphism, developmental delay, and ID. Associated multi-organ involvement can be malformations of Central Nervous System (88%), heart defects (71–75%), seizures (44–79%), skeletal anomalies (41%), vision problems, hearing loss, and other features [6].

The major phenotypic features observed in our cohort are summarised in Table 1.

Cardiac involvement in 1p36 can be classified into three major subgroups: CHD, CMP, and progressive aortic abnormalities. 

In fact, the most common cardiac abnormalities described in the literature in 1p36 deletion syndrome are the CHDs: ASD, VSD, PDA, valvular anomalies, TOF, and CoAo. In the present cohort, CHD was observed in 6/9 (66%), mainly septal defects (5/9), and in two patients (2/9) with more complex anatomy (patient 2 with hypoplastic left heart syndrome (HLHS) and patient 3 with Ebstein anomaly). 

Patients with CMP mostly had NCLV while a minor percentage reported DCM with end-stage heart failure (HF) [11]. In our cohort, NCLV was observed in 5/9 (55%) patients, with normal left ventricle (LV) function in one, mildly reduced LV function in three, and severe LV dysfunction in one patient. 

Genes that may contribute to the development of CHD and CMP associated with 1p36 deletions are *DVL1, SKI, RERE, PDPN, SPEN, CLCNKA, ECE1, HSPG2, LUZP1, WASPF2, SKI, PRKCZ, PRDM16, RERE, UBE4B*, and *MASP2*. 

**Figure 2:**
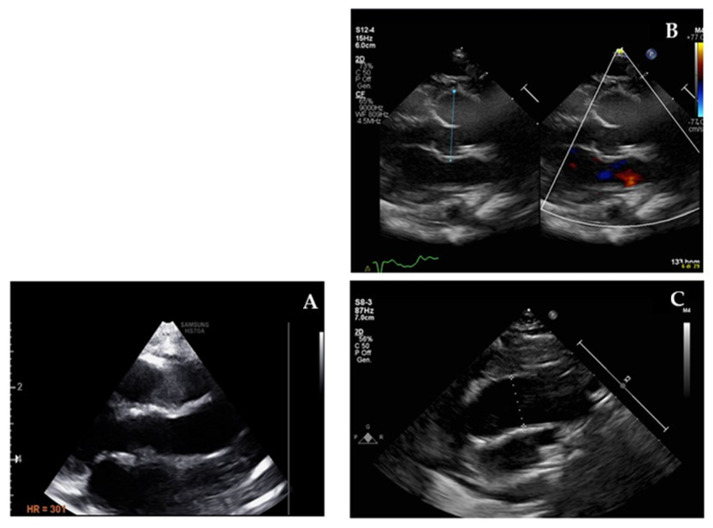
Echocardiographic imaging of normal ascending aorta (**A**) compared with patient 6 dilated aortic root (**B**) and dilated ascending aorta (**C**).

The third group including progressive aortic involvement represents the main issue of this paper since we describe three new patients (3/9 (33%)) who showed aortic dilatation in a normally tricuspid aortic valve. It was mild in two patients who were younger than 3 years old (patients 8 and 9) with potential progressive nature with time. Figure 2 shows the more severe aortic dilatation > 3 z-score that was observed in the third patient (patient 6). This makes up 33% of potential aortic involvement in a relatively limited cohort. During the follow-up period of our cohort, two patients (P6 and P9) showed slowly progressive aortic dilatation in the absence of haemodynamic or anatomic factors that can explain it. We opted to start medical treatment (Supplementary Materials 2). In the third patient (P8), no active follow up was available at our centre since the family moved to live in another region. Baseline echocardiography showed dilated aortic roots (Z-score 2.5) and ascending tracts with mild aortic regurgitation. This aspect is less described in the literature as being related to 1p36 deletion syndrome and might be underestimated due to its progressive nature, which might be overlooked since physicians recommend echocardiography at baseline visits (at the time of genetics diagnosis) and the imaging test might not be prescribed periodically. This issue might be a future emerging risk if adults with this rare condition represent a category that needs specific management in terms of medical—conservative or surgical—interventions. 

Thus far, only three patients with aortic involvement in 1p36 deletion are described in the literature. Campeau et al. described a patient with a tortuous aortic arch [14]. Zaveri et al. reported a mild aortic dilatation, but it was associated with BAV, which might represent a haemodynamic consequence [11]. Brazil et al. mentioned a female affected by dilated aortic root and pulmonary trunk in the association with PDA [15] (Table 2).

The 1p36-deleted region includes *SKI*, which is related to SGS (OMIM # 182212) in monogenic disorder. The main cardiovascular involvements that characterise this connective tissue disorder are mitral valve prolapse, dilation of the aortic root, vascular tortuosity, and aortic aneurysms [11].

SGS is a rare autosomal dominant disease with a marfanoid habitus and neurological, cardiovascular, and skeletal abnormalities. The protein encoded by *SKI* is a negative regulator of TGF-β signal;ing and appears to contribute to the determination of traits such as cleft lip/palate, skeletal abnormalities, progressive aortic involvement, and development delay in SGS [16,17]. Studies on dermal fibroblasts cultured from individuals with SGS confirmed the greater activation of TGF-β signalling cascades and the greater expression of target genes compared with control cells. TGF-β signalling is crucial for the normal development and maintenance of various organs, including the vascular system. SKI exerts a negative regulatory effect on TGF-β signalling by interacting with several proteins such as SMADs and other transcriptional co-regulators [18].

In this paper, we hypothesised a possible contribution of *SKI* in aortic abnormalities in 1p36 deletion syndrome. In fact, in our cohort, three patients with dilated aorta harboured deleted *SKI*. Patients 1, 2, and 3 had *SKI* deletion but did not show clear aortic involvement. However, regarding patient 2, we cannot completely exclude the aorto-myocardial “progressive nature” due to the fact that she had severe left-sided heart lesion (HLHS) and died at the age of 6 months old after prolonged hospitalisation and multiple palliative surgical procedures. Regarding patient 3, the last echocardiographic findings (at the age of 12 years old) showed mild mitral and aortic valve insufficiency in addition to Ebstein anomaly and LV function at the lower normal limit (ejection fraction (EF) 50%) with NCLV. In contrast, patient 1 did not have any signs of aortic involvement (last visit at the age 5 years old). 

This observation can be explained secondary to the rule of phenotypic variability in most genetic conditions. Moreover, aortic involvement in *SKI*-related SGS is often progressive in nature rather than congenital. Our patients might still be protected by the lower “expressivity” of the condition due to young age (last observation of pt 1: 5 years old, pt2: died at 6mth old, and pt 3: mild valvular insufficiency at 12 years old). In addition, it is known that SGS is characterised by a wide phenotypic variability where aortic root involvement is described in 34% of molecularly confirmed patients [12,16,17]. Therefore, we can hypothesise that, even in 1p36 deletion, the involvement of the aorta is variable and with an incomplete penetrance/age-related penetrance [6].

The 1p36 deletion causes a haploinsufficiency of *SKI* when harboured in the deleted region.

Literature reports explain that patients with the 1p36 deletion syndrome are haplo-insufficient for *SKI* and show some phenotypic overlap with SGS especially for hypotonia, ID, craniofacial dysmorphism (less craniosynostosis), vertebral abnormalities, and CHDs. Some suggested an explanation that distinguishing 1p36 deletion from SGS might be related to the involvement of contiguous genes in the 1p36 deletion syndrome and/or the putative dominant-negative potential of *SKI* variants in SGS, which might help retain their ability to form homo-dimeric complexes due to structural preservation of the *SKI*-interacting domain at their C-terminus, with functional deficits imposed by N-terminal mutations that selectively perturb R-SMAD and/or N-CoR interactions [7,17,19].

It is not possible to exclude a priori the involvement of multiple *SKI*-related mechanisms or molecular interactions with other genes in the deleted region in the aortic involvement of these patients. However, the fact remains that, when looking for aortic involvement in a limited cohort, one third of patients showed aortic dilatation. 

We admit the difficulties of this study due to the young age of our patients and limited number of the cohort; however, it is of interest to note that the only two patients in our series (P4 and P7) without cardiovascular involvement are those in whom the *SKI* was spared from the deleted chromosomal tract.

Patients with 1p36 deletion syndrome show some phenotypic overlap with SGS such as hypotonia, developmental delay, craniofacial dysmorphism, skeletal abnormalities, and cardiovascular abnormalities. However, traits such as dilation of the aortic root, vascular tortuosity, and aortic aneurysms typical of SGS are generally not “looked for regularly and periodically” in 1p36 deletion syndrome. At our centre, we established periodic cardiac screening in 1p36 deletion syndrome for monitoring of both myocardial and aortic features even in those with apparently normal anatomic findings. 

## 5. Conclusions

In consideration of the literature data and the results from our cohort, we might suggest the importance of periodic echocardiographic screening the aorta and arterial tree over time in clinical surveillance programs of patients with 1p36 deletion syndrome. It might be important to take into consideration a personalised approach for the specific medications suggested in TGF-β pathway defects that reduce hemodynamic stress on the aortic wall, such as β-blockers or ARBs, in patients with markedly dilated aortic root and/or ascending aorta.

Further studies, multicentric observations, and long-term cardiac surveillance in individuals who are older might be needed to determine the potential aortic and arterial involvement in 1p36 deletion syndrome 

## Figures and Tables

**Table 1:** A summary of the main cardiac (CHD, NCLV/CMP, and aortic involvement) and multisystemic features in our cohort of nine patients with 1p36 deletion syndrome.

	P1	P2	P3	P4	P5	P6	P7	P8	P9
Molecular genotype (hg19)	1p36.33p36.23(757,093–7,982,351)x1dn(7.2Mb),4q21.21q21.22(82,299,006–82,543,148)x3 pat(244Kb)	1p36.33p36.31(82154_6923634)x1(6.8Mb)	1p36.33p36.32(564424_4128574)x1dn(3.5Mb),Xp22.33p22.32(61091_5028407)x3 dn(4.9Mb)	1p36.33(82154_1258246)x1dn(1.2Mb), 1p36.33(1497824_2071340)x1dn(574Kb), 4p14(38857310_39367654)x3mat(510Kb)	1p36.33p36.32(82154_3441264)x1dn(3.4Mb)	1p36.33p36.22(82154_9600774)x1dn(9.5Mb)	1p36.33(82154_2098512)x1dn(2Mb)	1p36.33p36.32(82154_4418164)x1dn(4.3Mb)	1p36.33p36.31(82154_5514194)x1dn(5.4Mb), 2p23.1(30670973_31204981)x3 pat(534Kb)
Age at diagnosis/follow-up duration	3.6m/5.5ys	4.11m/22ds	11.2ys/1.3ys	5.10ys/1y	8.17m/4m	3.22m/1.6ys	5.2ys/-	4.2ys/1.7ys	4m/1.2ys
CHD	ASD	HLHS, Mitro-aortic hypoplasia, VSD, CoAo	Ebstein anomaly with TR, Mild MR and TR	No	ASD	ASD, VSD, PDA surgically repaired	No	No	ASD-OP, mild TR
CMP / NCLV	NCLV, EF 45%	No	NCLV, EF 50%	No, EF 67%	NCLV, severe DCMP EF 15%	NCLV, EF 65%	No	Mild LVH	NCLV, EF 45%, mild LVH
Aortic dilatation	Normal	Normal	Normal	Normal	Normal	Dilated aortic root, (Z-score 5), and ascending aorta (Z-score 2.9) In losartan therapy	Normal	Dilated aortic root (Z-score 2.5) and ascending tract with mild AR. Lost in F-UP	Dilated ascending aorta (Z-score 2.1); in ACEI and beta-blocker therapy
Growth delay	Yes	Yes	No	No, obesity	Lower normal limit	Yes	No	No	Lower limits
Epilepsy	Yes, Symptomatic Focal	No	Yes	No	No	Yes	No	Yes, West Syndrome	Yes, Focal
ID	Yes	Yes	Yes	Yes	Yes	Yes	Yes	Yes	Yes
Brain imaging	SEH	Cystic formations	Unknown	Unknown	Normal	Polymicrogyria	Unknown	Multiple periventricular heterotopic nodules	Polymicrogyria
Deafness	Bilateral asymmetric sensorineural	Unknown	No	No	No	Bilateral sensorineural	No	No	Bilateral sensorineural
Vision	Normal	Normal	Normal	Normal	Normal	Normal	Normal	Oscillatory nystagmus, dacryostenosis	Normal
Abdominal US	Bilateral renal pelvic dilatation	Monolateral renal pelvic dilatation	Hepatic steatosis	Unknown	Bilateral renal pelvic dilatation	Normal	Normal	Unknown	Gallbladder stones, dilated renal pelvis
Other malformations	Sacrococygeal fistula	Monolateral choanal stenosis	No	No	No	No	Phimosis	No	Normal
Other anomalies	Intermittent hypoglycaemia, abnormal thyroid tests	No	Scoliosis, lordosis	No	No	Chronic respiratory failure, feeding difficulties (PEG)	Allergies	No	Normal
Dysmorphisms	Marbled skin, very wide AF, splanchnocranial disproportion, upslanting palpebral fissures, sunken eyes, pointed nose, triangular chin, thin lips, triangular face, long fingers and toes, rocker bottom feet	Very large AF, bitemporal narrowing, prominent eyes, apparent hypertrophy of the inner side of the lip, long face, long fingers, irregular and deep dermatoglyphics	Hypotelorism, arched, eyebrows, prominent nostrils, high arched palat, thick lips, short neck, joint stiffness, long and tapered fingers, scoliosis, kyphosis, prominent abdomen and hepatomegaly, flat feet	Hypotelorism, upslanting palpebral fissures, high arched palate, short philtrum	Anomalous distribution of the masses in the axillary area (irregular folds), AF slightly broad, prominent frontal bulges, sunken orbits, short nose, broad tip, small lips with downward angles, high arched palate, small low-set ears, bilaterally hands showed a peculiar aspect of transverse “line” in the terminal part of the metacarpals, appearance of skin syndactyly at the base of the fingers	Relative macrocephaly, turricephaly, AF >> 3x3cm, frontal bossing, thin palpebral fissures, short nose with flattened root, prominent columella, thin upper lip, dysmorphic appearance of the palate, gingival hypertrophy, right “crumpld ear”, short neck, narrow chest, left hand single palmar crease, rectus diastasis, small umbilical hernia, moderate left inguinal hernia, limbs showed fixed contractures of the flexors of the knees and the feet with a club feet appearance	Hypotelorismo, deep set eyes, angles of the mouth downturned	Depressed nasal bridge and of the frontal orbital tract, hypotonia, ligamentous laxity	Flat facies, depressed nasal root, puffy eye appearance, posterior cleft palate, micrognathia, dysmorphic auricles

Abbreviations: AF, anterior fontanel; AR, aortic regurgitation; ASD, atrial septal defect; CHD, congenital heart defect; CMP, cardiomyopathy; CoAo, Coarctation of the aorta; DCMP, dilated cardiomyopathy; EF, ejection fraction; NCVL, noncompaction of left ventricle; HLHS, hypoplastic left heart syndrome; ID, intellectual disability; LVH, left ventricular hypertrophy; MR, mitral regurgitation, OP, ostium primum; PDA, patent ductus arteriosus; PEG; Percutaneous endoscopic gastrostomy; SHE, sub-ependymal haemorrhage; TR, tricuspid regurgitation; US, ultrasound; VSD, ventricular septal defect.

**Table 2:** A summary of the review from the literature of previously described patients with deletion 1p36 and aortic involvement.

Reference and Patient Identification	Start-Stop (hg19)	Size (Mb)	Heredity	CHD (Co-Existing Cardiomyopathy)	Other
Campeau et al. 2008, Patient 1 [14]	1–10247416	10.2	de novo	Asymmetric ventricles, muscular VSD, tortuous aortic arch, PDA	Hypotonia, single febrile seizure, bilateral colpocephaly, moderate to severe non-obstructive hydrocephalus, sensorineural hearing loss, short femurs, unilateral club foot, submucous cleft palate, velopharyngeal incompetence, dysmorphic features
Zaveri et al. 2014, Patient 2 [11]	1–3581432	3.6	de novo	bicuspid aortic valve, mild aortic dilatation	Developmental delay, mild unilateral conductive hearing loss, concern for seizures
Brazil et al. 2014, Patient 18 [15]	na	na	na	PDA, dilated aortic root and pulmonary trunk	N/A

## Supplementary Materials

The following are available online. A case series description is available online. A case series description is in Supplementary Material.
