# Supplementary material for: 1p36 Deletion Syndrome and the Aorta: A Report of Three New Patients and a Literature Review
Source: J Cardiovasc Dev Dis. 2021 Nov 19;8(11):159. doi: 10.3390/jcdd8110159 (PMC8618808; doi:10.3390/jcdd8110159)
Supplement: Supplementary file 1 [file jcdd-08-00159-s001.zip › jcdd-1380717-supplementary.pdf]

Journal of
*Cardiovascular
Development and Disease*

Article

1p36 Deletion Syndrome and the Aorta: A Report of Three New Patients and Literature Revision

Supplementary Material

1. Patients

1.1. Patient 1 (P1), female

The proband is the daughter of unrelated parents. The mother's first two pregnancies resulted in two spontaneous abortions (6th and 14th weeks of gestation). Her two elder brothers are in good health. The pregnancy went well until the fifth month when there was suspected cardiac ventricular disproportion. She was born at 37 + 3 weeks by Caesarean section for intrauterine growth restriction (IUGR). Her birth parameters were a weight of 1.7Kg (<3°), a length of 43cm (<3°), and an occipitofrontal circumference (OFC) of 30cm (<3°). At birth, there was suspicion of coarctation of the aorta (CoAo) that was excluded. Echocardiography showed an atrial septal defect (ASD) (which was closed spontaneously), noncompaction of the left ventricle (NCLV), and progressive ventricular dysfunction (ejection fraction (EF) 45%). Her aortic root diameter was normal (her last echocardiography was at 5 years old). Heart failure (HF) is managed with anti-HF medications with significant improvement. Brain ultrasound (US) showed signs of sub-ependymal haemorrhage and dilatation of the lateral cerebral ventricles. Other clinical features included symptomatic focal epilepsy, intellectual disability (ID), bilateral asymmetric sensorineural deafness, sacro-coccygeal fistula, and bilateral renal pelvic dilatation. Blood tests showed intermittent hypoglycaemia, increased LDH, and abnormal thyroid tests. A physical examination at 5years and 4 months confirmed microsomy (weight 13Kg and height 104cm), splanchno-cranial disproportion, upslanting palpebral fissures, triangular face, sunken-eyed, pointed nose, thin lips, a triangular chin, poorly represented subcutaneous tissue, marbled skin, long fingers and toes, and rocker bottom feet.

Genotype: 1p36.33p36.23(757,093-7,982,351)x1 dn (7.2Mb),
4q21.21q21.22(82,299,006-82,543,148)x3 pat (244Kb)

1.2 Patient 2 (P2), female

Prenatal US showed hypoplastic left heart syndrome (HLHS) that was confirmed at birth when the echocardiography showed mitro-aortic hypoplasia, severe left ventricular (LF) hypoplasia, multiple ventricular septal defects (VSDs), anterograde aortic flux, CoAo with aortic arch hypoplasia, and persistent left superior vena cava. Her birth parameters were weight 2.3Kg (3°), length 47cm (9°), and OFC 31cm (<3°). A univentricular palliative procedure was undertaken but never reached second-stage palliation since she remained at stage I: hybrid procedure, damus kaye stansel (DKS). She continued to present severe hemodynamic instability and was on extracorporeal membrane oxygenation (ECMO) when multiorgan failure developed. She died at the age of five months in severe failure to thrive (Cachexia). Multiorgan screening included a brain US that did not show any malformation except for cystic formations, monolateral choanal stenosis, and mild left renal calico-pelvic ectasia. Her eye

visit was normal. A physical examination showed severely dystrophic appearance, long face, very large anterior fontanel, bitemporal narrowing, prominent eyes, apparent hypertrophy of the inner side of the lip, long fingers, and irregular and deep dermatoglyphics.

Genotype: 1p36.33p36.31(82154_6923634)x1 dn (6.8Mb)

1.3 Patient 3 (P3), female

She is the daughter of unrelated parents. The mother had six spontaneous abortions. She was born at term by Caesarean section at 36 weeks for placental abruption. At birth, her weight was 2.4Kg (3°) and her length was 45cm (3°).

At birth, the neonatologists revealed heart murmur, for which echocardiography was performed, showing mild Ebstein anomaly of the tricuspid (the septal flap was only slightly displaced caudal to the valvular plane) with mild valvular regurgitation. The mitro-aortic regurgitation was minimal. Her left ventricle (LV) function was at lower limits (EF 50%). Marked mid-apical and lateral ventricular hyper-trabeculation, which satisfies the criteria of noncompact myocardium with a noncompact/compact (NC/C) ratio> 2 and a normal aortic root diameter. Her vital signs showed intermittent hypertension. Other clinical features included ID, epilepsy, severe hepatic steatosis (steatohepatitis on biopsy), hyper-transaminasemia, right-convex lumbar scoliosis and lordosis, cervico-diaphyseal valgus of the femoral necks with lower limbs asymmetry (5.6 mm), and bilateral flat feet. A physical examination at last visit (11years and 6months) showed the following: weight 45Kg (75°), height 154cm (75°), OFC 50.5cm (3-25°), hypotelorism, arched eyebrows, prominent nostrils, a high arched palate, thick lips, a short neck, joint stiffness, long and tapered fingers, scoliosis, kyphosis, flat feet, a prominent abdomen, and hepatomegaly.

Genotype:arr[GRCh37]1p36.33p36.32(564424_4128574)x1 dn (3.5Mb),Xp22.33p22.32(61091_5028407)x3dn (4.9Mb). The in-depth analyses using the FISH technique on the cytogenetic preparations of the proband and of the parents made it possible to associate the results found in the presence of a rearranged chromosome 1, derived from an unbalanced translocation occurring de novo between the short arm of a chromosome 1 and the short arm of an X chromosome.

1.4 Patient 4 (P4), male

The proband is the son of healthy unrelated parents. He was born at term, after uneventful pregnancy, from vaginal delivery. At birth, his weight was 3.250Kg (40°), his length was 50cm (50°), and his OFC was 33cm (9°). His APGAR score was 9-10. He had a normal neonatal period and no convulsions. Echocardiography showed normal anatomy and functional parameters (EF 67%), and normal aortic diameters. At birth, hypotonia was noticed. Later, obesity and ID were noticed. A physical examination at the age of 7 years showed macrosomy (weight 52.10Kg (>97°), height 136.50cm (>97°), and OFC 54cm (>97°)), ID, simple language, hyperactivity, hypotelorism, upslanting palpebral fissures, a high arched palate, a short philtrum, and mild benign hypermobility.

Genotype: arr[GRCh37] 1p36.33(82154_1258246)x1 dn (1.2Mb), 1p36.33(1497824_2071340)x1 dn (574Kb), 4p14(38857310_39367654)x3 mat (510Kb)

1.5 Patient 5 (P5), female

She is the only child of unrelated parents. The mother had a previous twin pregnancy that was terminated spontaneously at the end of the first trimester. She was born at 36+4 weeks of gestation by Caesarean section. Her birth parameters were a weight of 1.99Kg (2°) and a length of 43cm (<3°). She thrived well until the age of 7 months when her parents started to notice feeding difficulties and continuous irritability. Echocardiography showed severe dilated cardiomyopathy (EF 15%) based on NCLV, but her aortic diameters were normal. She had hypotonia, but her brain US was normal. Bilateral renal pelvic dilatation was minimal. A physical examination at the age of 14 months showed the following parameters: weight 8.725Kg (5°); length 72cm (3°); OFC 43cm (3°); anomalous distribution of the subcutaneous tissue, in particular, in the axillary region (irregular folds); prominent bossing; sunken eyes; short nose with a broad tip; small lips with downward angles; a high arched palate; and small low-set ears. Bilaterally, her hands showed a peculiar aspect of a transverse "line" in the terminal part of the metacarpals, creating an appearance of skin syndactyly at the base of the fingers.

Genotype: arr[GRCh37] 1p36.33p36.32(82154_3441264)x1dn (3.4Mb)

1.6 Patient 6 (P6), male

He is the child of consanguineous parents (high inbreeding) from Morocco. His family history is negative for CHDs, ID, genetic syndromes, and sudden death at young age. The proband has three healthy elder brothers.

The pregnancy was relatively uneventful with reduced active foetal movements. He was born at term by urgent Caesarean section for breech presentation. At birth, his weight was 2.7Kg (9°pc) and he had severe asphyxia, a right clavicular fracture, a left clubfoot, and pre-sacral dimples.

At birth, echocardiography showed ASD, severe left atrial and ventricular dilatation but with preserved EF, large subaortic VSD, PDA, and mild mitral and tricuspid regurgitations. At the age of 40days old, he was surgically operated for PDA and septal closure. However, the echocardiographic findings at the age of 5months old showed a progressive nature in terms of severe dilatation at 1.7 cm of the aortic root and dilatation of the ascending aorta. These data were confirmed by computer tomography (CT) scan, which confirmed dilatation of the sino-tubular junction (1.4 cm, Z-score 3.5), aortic root dilatation (20x20 mm, Z-score 5), and ascending aorta (1.4 cm, Z-score 2.9). He was managed with Losartan with stable aortic diameters (1-year follow-up). Moreover, the echocardiography showed LV hyper-trabeculation but normal EF 65%.

At 2 months old, he showed drug-resistant multifocal epilepsy. Brain magnetic resonance imaging (MRI) showed a partial increase in cortical thickness, polymicrogyric appearance of the posterior parietal and sylvian regions, reduced white matter expression in the subcortical and periventricular zones, and marked hypoplasia of the main interhemispheric commissures associated with enlargement of the supratentorial ventricular system and the cerebrospinal fluid (CSF) spaces. Moreover, he had ID, bilateral sensorineural deafness, chronic respiratory failure manged by non-invasive ventilation (NIV), and feeding difficulties that needed percutaneous endoscopic gastrostomy (PEG).

A physical examination at the age of six months showed a weight of 4.023Kg (<3°), a length of 61cm (<3°), an OFC of 39cm (<3°), relative macrocephaly with turricephaly, fontanel >> 3x3cm, frontal bossing, thin palpebral fissures, short nose with flattened root, prominent columella, thin upper lip, dysmorphic appearance of the palate, gingival hypertrophy, a right "crumpled ear", a short neck, a narrow chest, rectus diastasis, small umbilical hernia, moderate left inguinal hernia, and left-hand single palmar crease. The limbs showed fixed

contractures of the flexors of the knees and the feet with a club feet appearance. The growth parameters at 19 months were a weight of 9 Kg, a length of 85 cm, and an OFC of 45 cm.

Genotype: arr[GRCh37] 1p36.33p36.22(82154_9600774)x1 dn (9.5Mb)

1.7 Patient 7 (P7), male

The only child of healthy non-consanguineous parents. He was born at term by Caesarean section. His birth weight was 3.1Kg (25°). He was admitted for the first time at the age of 11 months for multiple food anaphylactic reaction (allergic to milk and wheat protein). His weight was 9.380Kg (50°), his length was 72cm (15°), and his OFC was 46cm (50°). His eye visit was normal. He showed phimosis. Echocardiography was performed at another institute and reported normal finding (no aortic dilatation). Due to the presence of dysmorphic features and ID, genetic investigations were performed, showing arr[GRCh37] 1p36.33(82154_2098512)x1 dn (2Mb)

1.8 Patient 8 (P8), male

The proband is the son of healthy non-consanguineous parents. He was born at term by Caesarean section due to a lack of engagement. At birth, his weight was 2.350Kg (3°), his length was 49cm (32°), his OFC was 34cm (36°), and his APGAR score was 7-8. Due to the presence of delayed developmental milestones, the child was admitted to our hospital, where a focal symptomatic epilepsy and west syndrome were diagnosed. A brain MRI showed small fusiform areas in the supra and paratrigonal areas, more likely compatible with mild district ectasia of regional perivascular spaces, and multiple periventricular heterotopic nodules. A physical examination showed hypotonia, hypermobility of joints, facial dysmorphism with oscillatory nystagmus, dacryostenosis, and a depressed nasal bridge. He had abnormal visual-evoked potentials. Echocardiography showed mild LV hypertrophy, mild ectasia of the aortic root (Z-score 2.5), and an ascending tract with mild aortic regurgitation.

Genetic investigations showed arr[GRCh37] 1p36.33p36.32(82154_4418164)x1 dn (4.3Mb)

1.9 Patient 9 (P9), female

The proband is the daughter of healthy non-consanguineous parents. She was born at home at 41+4 weeks gestation, with a weight of 2.850kg (15°) and an APGAR score of 8-9. At birth, a posterior cleft palate, hypothermia, severe mixed metabolic acidosis (pH 6.97), and perinatal asphyxia were observed. She was admitted to our hospital, where symptomatic focal epilepsy was diagnosed. A brain MRI showed an increase in the size of the supratentorial ventricular system, mild subependymal haemorrhages, and micropolygyria of the cerebral cortex in the frontal lobe (right> left). A hearing evaluation confirmed bilateral deafness. An abdominal ultrasound showed gallbladder stones and dilated renal pelvis. Echocardiography showed disynergic movement of the interventricular septum, hypertrophic appearance of both ventricles, mild tricuspid insufficiency, "ostium primum" ASD, hyper-trabeculation of the apical and lateral walls of the left ventricle with NC/C> 2 confirming NCLV, LV dysfunction (EF 45%), and dilated left ventricle (z-score 2.4). Moreover, echocardiography, since age 6 months, showed mild dilatation of the ascending aorta at 1.3cm (z-score 2.1) but not of the root at 1.4 cm (z-score 1.3). She was managed with an ACE-inhibitor and beta-blocker therapy. A physical examination at last visit (age 11 months) showed cleft palate, micrognathia, flat facies with depressed nasal root, a puffy eye appearance, and dysmorphic

auricles. Genetic investigations showed 1p36.33p36.31(82154_5514194)x1 dn (5.4Mb), 2p23.1(30670973_31204981)x3 pat (534Kb).

**Table S1. Summary of the echocardiographic findings of patients 6 and 9.**		
	**Patient 6**	**Patient 9**
**Cardiovascular findings**	ASD, subaortic VSD, at 5mth aortic root dilatation, STJ, and ascending aorta	ASD OP, Mild IT, NCLV, Mild LV dysfunction, dilatation of the aortic root, and ascending aorta
**Age at diagnosis of aortic abnormalities**	5 months	12 days
**Aortic root (cm) at time of diagnosis**	1.7	1.2
**Z-score**	5.1	1.2
**Age at first F-UP**	9 months	11 months
**Aortic root (cm) in first F-UP**	1.8	1.4
**Z-score**	3.1	0.42
**Age at second F-UP**	13 months	
**Aortic root (cm) in second F-UP**	1.9	
**Z-score**	3	
**Age at third F-UP**	22 months	
**Aortic root (cm) in third F-UP**	2.1	
**Z-score**	4.2	
**Ascending aorta (cm) at time of diagnosis**	1.5	1.1
**Z-score**	4.6	2.1
**Ascending aorta (cm) in first F-UP**	1.6	1.4
**Z-score**	4.8	1.3
**Aortic root (cm) in second F-UP**	2	
**Z-score**	5.2	
**Ascending aorta (cm) in third F-UP**	2	
**Z-score**	3	
**Treatment**	Losartan: 3mg x1	Carvedilolo 0,2 mg x 2<br>Enapren 0,4 mg x 2

Abbreviations: ASD, atrial septal defect; F-UP: Follow Up; IT, tricuspid valve insufficiency; LV, left ventricle; NCLV, noncompaction of left ventricle; OP, ostium primum; STJ, sinotubular junction; VSD, ventricular septal defect
